# Evaluation and use of disaccharides as energy source in protein-free mammalian cell cultures

**DOI:** 10.1038/srep45216

**Published:** 2017-03-30

**Authors:** Dawn Sow Zong Leong, Janice Gek Ling Tan, Christine Lin Chin, Shi Ya Mak, Ying Swan Ho, Say Kong Ng

**Affiliations:** 1 Bioprocessing Technology Institute, Agency for Science, Technology and Research (A*STAR), Singapore

## Abstract

Mammalian cells are generally considered to be unable to utilize polysaccharides for cell growth because the phospholipid bilayer in the cell membrane has very low permeability to sugars. With the recent discovery of the only known animal disaccharide transporter, a sucrose transporter, we considered the potential use of polysaccharides as energy source, because that can impact biopharmaceutical manufacturing by potentially increasing carbohydrate loading in the culture medium and decreasing lactate accumulation. In this study, we found that mammalian cells can utilize maltose for growth in the absence of glucose and successfully adapted CHO-K1, CHO-DG44 and HEK293 cells to grow in glucose-free, maltose-containing serum-free protein-free media. We then cultivated a non-adapted CHO-K1 producer cell line in media containing both glucose and maltose to show that the cells can utilize maltose in a biphasic manner, that maltose enters the cells, and that maltose utilization only took place in the presence of the cells. This is the first report of a protein-free mammalian cell culture using a disaccharide as energy source.

Mammalian cells are chemoheterotrophic and typically require a carbohydrate source for growth in cultures. As carbohydrates have low permeability through the phospholipid bilayer that makes the bulk of the cell membrane^1^,^2^, sugar transport into the cell is facilitated by transporter proteins^3^,^4^,^5^. Hence, for the cultivation of mammalian cells, glucose is the single most commonly used carbohydrate, because it can be efficiently transported into the cells through two major families of monosaccharide transporters, the sodium-glucose linked transporters (SGLT)^4^ and glucose transporters (GLUT).

In addition to glucose, other carbohydrate sources have been tested for their ability to support growth of animal cell cultures^6^,^7^. In these reports, monosaccharides galactose, fructose and mannose were demonstrated to be utilized by most cell types in both serum and serum-free culture media, consistent with the availability of transporter proteins to internalize these sugars^8^,^9^. Polysaccharides had also been shown to support cell growth in cell cultures supplemented with serum, because serum contains saccharidases that were essential for the breaking down of the complex carbohydrates in the culture media^7^. In another study, heat inactivated serum devoid of amylase and/or maltase activities and culture dishes coated with serum-containing medium were used to isolate Chinese Hamster Ovary (CHO) cell variants that can utilize maltose or starch^10^. The authors showed that the culture dish coated with serum-containing medium did not contribute to saccharidase activity, and thus they hypothesized that endogenous carbohydrate hydrolases, otherwise only expressed in the small intestines, were induced in these isolates to allow for their growth in maltose and starch-containing media^10^. Nonetheless, saccharidase-containing serum was used in this study to coat the culture dishes, and how this contributed to cell utilization of maltose and starch was not evaluated.

To our knowledge, there is no report to-date on the use of polysaccharides to support cell growth in serum-free cultivation of mammalian cells, even though serum-free and protein-free cultivation of mammalian cells has been reported since the 1970s and 1980s respectively^11^,^12^. This is not surprising, since there is only one known animal disaccharide sucrose transporter that was recently reported^13^. Whether polysaccharides can support mammalian cell growth in serum-free culture is of interest for both basic and applied sciences. For the basic understanding of mammalian cell metabolism of polysaccharides, the use of serum-free culture can completely preclude the role of saccharidase from serum contributing to the survival of cells utilizing only polysaccharides, which could not be ruled out in the previous report^10^. If a serum free mammalian cell culture utilizing polysaccharides is obtained, the culture can be a model to elucidate yet unknown mechanisms of polysaccharide transport and metabolism in mammalian cells, such as the recent discovery of the first known animal sucrose transporter in *Drosophila melanogaster*^13^.

In this study, we evaluated the use disaccharides, the simplest polysaccharides, to support the growth of a mammalian cell line in a serum-free protein-free culture. CHO and Human Embryonic Kidney 293 (HEK293) cells were chosen for the potential practical application of the study, since these are two of the most widely used mammalian cell line for the manufacture of recombinant protein therapeutics. The growth of these cells in disaccharide-containing media was then characterized.

## Results

### Evaluation of disaccharides to support growth of CHO and HEK293 cells

**Figure 1:**
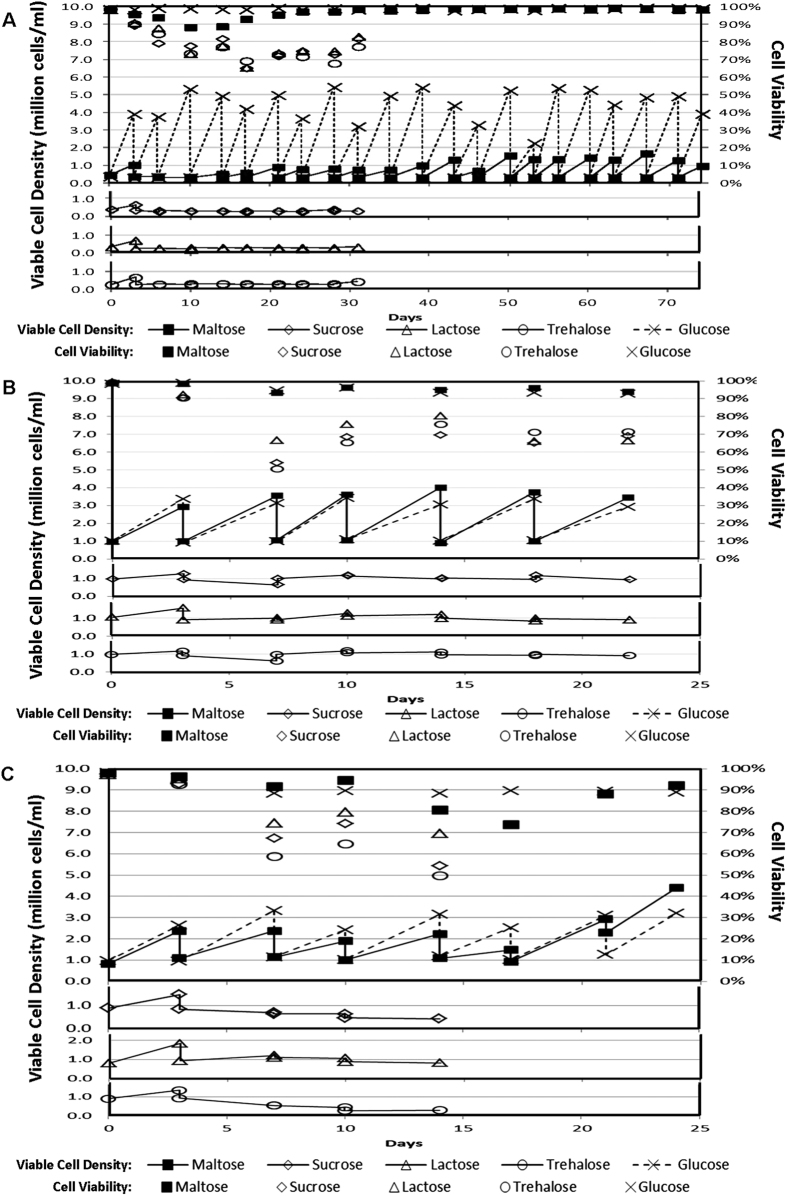
Adaptability of Chinese Hamster Ovary (CHO) cells and HEK293 cells in various disaccharide media. (**A**) CHO-K1 cells were cultivated in a serum-free protein-free cell culture medium, HyQ PF-CHO, with 3.6 g/l of different sugars (maltose, sucrose, lactose, trehalose or glucose) as carbohydrate source. The viable cell densities (lined markers) and culture viabilities (marker only) of these cultures at the beginning and end of each passage over a period of 74 days were plotted. Cultures with sucrose, lactose or trehalose were terminated on Day 31 due to the decreased culture viabilities and reduced viable cell densities. The experiment was repeated with a seeding cell density of 1.0 × 10^6^ cells/ml to obtain similar results. (**B**) CHO-DG44 cells were cultivated with a seeding cell density of 1.0 × 10^6^ cells/ml in a serum-free protein-free cell culture medium, HyQ PF-CHO, with 10 g/l of different sugars (maltose, sucrose, lactose, trehalose or glucose) as carbohydrate source. The viable cell densities (lined markers) and cell viabilities (marker only) of these cultures at the beginning and end of each passage over a period of 22 days were plotted. (**C**) HEK293 cells were cultivated with a seeding cell density of 1.0 × 10^6^ cells/ml in a protein-free chemically defined cell culture medium, PFCDM, with 10 g/l of different sugars (maltose, sucrose, lactose, trehalose or glucose) as carbohydrate source. The viable cell densities (lined markers) and cell viabilities (marker only) of these cultures at the beginning and end of each passage over a period of 22 days were plotted. Cultures with sucrose, lactose or trehalose were terminated on Day 14 due to the decreased culture viabilities and reduced viable cell densities.

To evaluate the use of disaccharides to support the growth of mammalian cells, a CHO cell line, CHO-K1, was cultivated using a seeding cell density of 0.3 × 10^6^ cells/ml, with 3.6 g/l of maltose, sucrose, lactose, trehalose or glucose as energy source in a serum-free protein-free cell culture medium HyQ PF-CHO. Osmolality of these culture media were determined to be between 308 and 324 mOsm/kg, well within the range for optimal mammalian cell culture. The viable cell densities and culture viabilities of these cultures at the beginning and end of each passage over a period of 74 days are shown in Fig. 1A. While the cells in glucose containing medium grew to high culture viabilities and viable cell densities at each passage, those parameters for cells in disaccharide media decreased and remained stagnant respectively. However, culture viabilities and viable cell densities of the maltose culture started to pick up on Day 14. Proliferation of the cells in maltose culture then maintained over the period of the time studied, while those in the other disaccharides were terminated on Day 31 due to the lack of cell growth and depressed culture viabilities. The same experiment repeated using a higher seeding cell density of 1.0 × 10^6^ cells/ml using CHO-K1 cells (data not shown), CHO-DG44 cells (Fig. 1B) and HEK293 cells in DMEM/F12-based Protein-Free Chemically Defined Media (PFCDM) (Fig. 1C) gave similar results. Hence, we demonstrated for the first time that mammalian cells can proliferate in serum-free protein-free culture medium utilizing maltose, but not sucrose, lactose or trehalose, as sugar source.

### Growth and biochemical profiles of CHO-K1 cells adapted to grow in maltose culture medium

**Figure 2:**
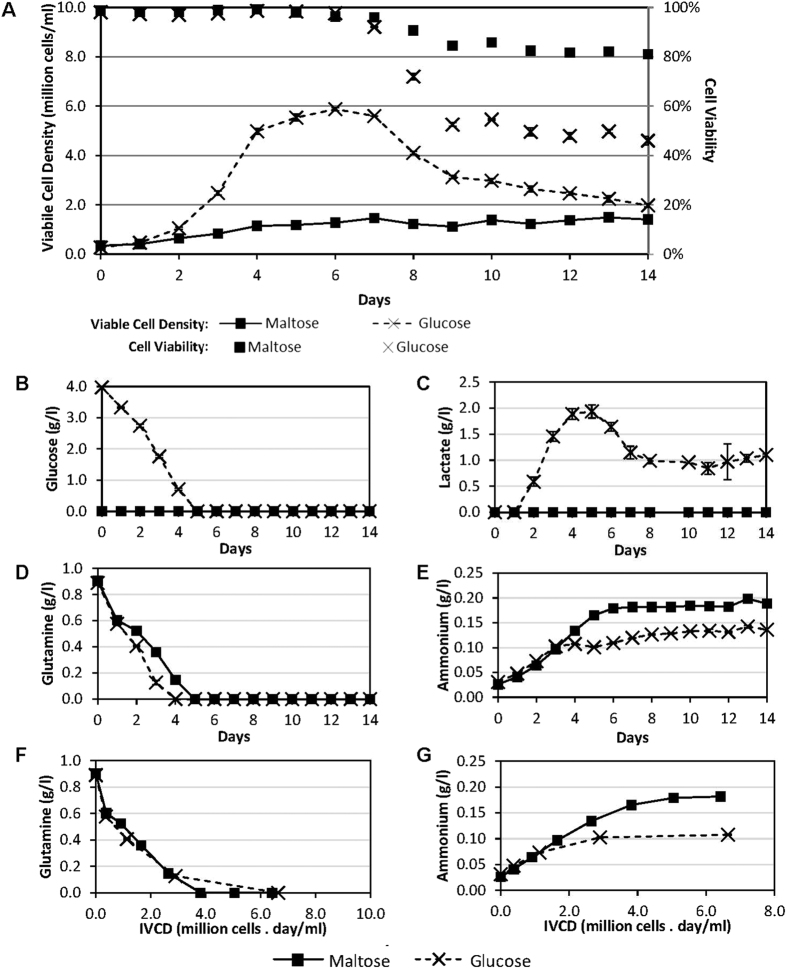
Growth and biochemical profiles of CHO-K1 cells adapted to and cultivated in maltose medium, compared to non-adapted CHO-K1 cells cultivated in glucose medium. CHO-K1 cells that were pre-adapted to a serum-free protein-free cell culture medium, HyQ PF-CHO with 3.6 g/l of maltose as carbohydrate source (square marker) and normal non-adapted CHO-K1 cells cultivated in the same culture medium but with glucose as the carbohydrate source (cross) were monitored over 14 days, to obtain their (**A**) Viable cell densities (lined marker) and culture viabilities (marker only), and (**B**) Glucose, (**C**) Lactate, (**D**) Glutamine and (**E**) Ammonium profiles. To illustrate the specific consumption and production rates of (**F**) Glutamine and (**G**) Ammonium, the concentrations of these components were also plotted against integral viable cell density (IVCD). The averages and standard deviations from 3 replicate shake flasks were plotted.

To validate the observation, the growth profile of the adapted CHO-K1 cell line in the maltose protein-free medium was then compared to the CHO-K1 cells cultivated in the glucose protein-free medium, using similar initial cell density and sugar concentration of 0.3 × 10^6^ cells/ml and 3.6 g/l respectively (Fig. 2). The cells cultivated with maltose as energy source grew to a much lower maximum viable cell density of 1.4 × 10^6^ cells/ml on Day 7 compared to that with glucose which reached 5.9 × 10^6^ cells/ml on Day 6 (Fig. 2A). As the initial sugar concentration was the same, we postulate that this difference in maximum viable cell density may be partly due to the depletion of other nutrients such as glutamine, since glutamine was depleted at similar rates till completely utilized on Days 4 and 5 for the glucose and maltose cultures respectively (Fig. 2D). Using viable cell density data till Day 4, the doubling time of the maltose culture was 53.7 h compared to 22.3 h for the glucose culture, suggesting that the rate of energy metabolism may be limiting the cell growth rate in the maltose culture. Interestingly, the maltose culture maintained at high culture viabilities greater than 80% over a longer period of time, compared to cells cultivated in glucose (Fig. 2A). This may be due in part to the lower maximum cell density that the maltose culture reached, which in turn may have resulted in higher amounts of un-metabolized nutrients and a lower accumulation of toxic metabolites at later time points of the culture. Another reason may be that the cells in the maltose culture had a lower metabolism due to the limiting energy uptake rate, and this resulted in less cellular stress and slower cell death.

Examining critical biochemicals in the culture medium, glucose was not detectable in the maltose culture (Fig. 2B), confirming the lack of glucose in the medium from the onset of the culture. As glucose concentration maintained at an undetectable level throughout the culture, this suggests that maltose was not hydrolyzed into glucose in the culture medium by secreted maltases, but was consumed by the cells. This consumption may have occurred via one of the 3 possible mechanisms: (1) maltose may be transported into the cells prior to hydrolysis by intracellular maltase such as acid alpha glucosidase (GAA), or (2) maltose may be hydrolysed by plasma membrane bound maltase, such as intestinal maltase-glucoamylase (MGAM) and sucrase-isomaltase (SI), and immediately taken up by the glucose transporters that are also found on the plasma membrane. These additional steps for energy metabolism may be rate limiting to result in the slow growth rate of the maltose culture, as discussed above. The lack of lactate production in the maltose culture (Fig. 2C) supports this hypothesis as it suggests that the cells in the maltose culture utilized the more energy efficient Krebs cycle, rather than ending the glycolysis pathway with lactate production, as embodied by the lactate generation in the glucose culture (Fig. 2C).

While glutamine consumption was slower in the maltose culture compared to the glucose culture (Fig. 2D), it was likely due to the slower growth of the maltose culture. This is verified by a comparison of the specific glutamine consumption of the glucose and the maltose cultures: By plotting the glutamine profiles of the cultures against the IVCD, it was observed that the specific glutamine consumption, given by the slope of the curve, is similar between the two cultures (Fig. 2F). This demonstrates that glutamine consumption rate of each cell was unaffected by the use of maltose. Given the slower growth rate and lower maximum cell density of the maltose culture, the similar specific glutamine consumption rate suggests that more glutamine in the maltose culture was being channeled into energy metabolism instead of cellular replication, when compared to the glucose culture. This hypothesis is supported by the ammonium production profiles: while the specific ammonium production rate was initially similar for both cultures, that for the glucose culture slowed considerably from Day 2 (Fig. 2G) to attain a final ammonium concentration of 0.14 g/l (Fig. 2E), whereas the specific ammonium production rate of the maltose culture continued at a similar rate till Day 5 to attain a higher final ammonium concentration of 0.20 g/l. The slower specific ammonium production rate of the glucose culture from Day 2 can be attributed to the higher cell growth rate (Fig. 2A) and a similar ammonium production rate, suggesting that more amino acids were utilized for cell replication compared to the maltose culture. The difference in the final ammonium concentrations further supports this, as it suggests that more amino acids are subjected to de-amidation to supplement the energy consumption of the cells in the maltose culture when compared to the glucose culture, since both cultures have the same starting concentration of glutamine and other amino acids. We also noted that the time at which ammonium production plateaued, at Day 4 and Day 6 for the glucose and maltose cultures respectively, corresponded with the times of glutamine depletion for the respective cultures, suggesting the ammonia accumulation may be partly due to spontaneous glutamine deamidation.

### Application of maltose to sustain culture viability upon glucose depletion

Since CHO cells can utilize maltose as energy source and maintain high cell culture viability for extended periods in protein-free culture medium containing maltose in the absence of glucose (Fig. 2), we proceeded to evaluate whether maltose can be utilized to sustain the culture viability of a non-adapted CHO-K1 production cell line upon glucose depletion. The cells were cultivated in a medium containing both glucose and maltose, to examine whether the cells can grow using glucose in the medium for normal cell growth, followed by a switch to maltose metabolism to maintain culture viability and protein production. This will potentially extend the application of maltose supplementation to non-adapted cell lines, and also be a simple method to extend cell culture viability for higher recombinant protein productivity in batch cultures.

**Figure 3:**
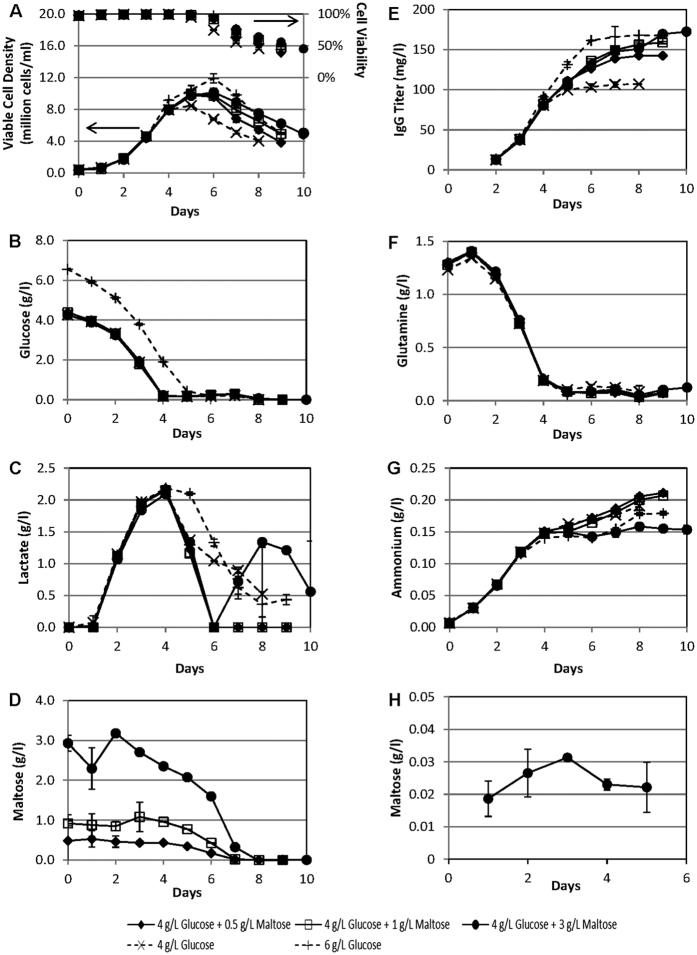
Growth and biochemical profiles of a recombinant monoclonal antibody producing CHO-K1 cell line (SH87) cultivated in protein-free chemically defined medium (PFCDM) with both glucose and maltose as sugar source. SH87 cell routinely maintained in glucose-only PFCDM was sub-cultivated into PFCDM with 4 g/l glucose, 4 g/l glucose + 0.5 g/l maltose, 4 g/l glucose +1 g/l maltose, 4 g/l glucose + 3 g/l maltose, or 6 g/l glucose. The cultures were monitored till culture viabilities were lower than 50%, to obtain their (**A**) Viable cell densities (lined marker) and culture viabilities (marker only), and (**B**) Glucose, (**C**) Lactate, (**D**) Maltose, (**E**) IgG titer, (**F**) Glutamine, and (**G**) Ammonium concentrations in the culture media. (**H**) SH87 cells sub-cultivated in PFCDM with 4 g/l glucose, or 4 g/l glucose + 3 g/l maltose, were harvested from Days 0 to 5 for LC-MS quantification of intracellular maltose concentrations. Intracellular maltose was found to be absent (below detection limit) in all samples obtained from the 4 g/l glucose cultures and in the sample from Day 0 of the 4 g/l glucose +3 g/l maltose cultures. The averages and standard deviations of 2 technical replicates from one set of shake flasks were plotted for (**D**). For the other profiles, the averages and standard deviations from 2 replicate shake flasks were plotted.

For this evaluation, we used a CHO-K1 cell line (SH87) that is producing an anti-Her2 monoclonal antibody^14^. Batch shake flask cultures of these cells in PFCDM with 4 g/l glucose, 6 g/l glucose, or 4 g/l glucose supplemented with different concentrations of maltose were monitored till culture viabilities dropped below 50% (Fig. 3). 4 g/l glucose was chosen as the base glucose concentration because this will allow for a premature but controlled cell growth limitation that is mainly due to glucose depletion in the batch cultures for this PFCDM. This controlled growth limitation is observed in Fig. 3A where the 4 g/l glucose culture reached maximum viable cell density (VCD) of 8.5 × 10^6^ cells/ml on Day 5, one day earlier than the 6 g/l glucose culture, which reached maximum VCD of 11.9 × 10^6^ cells/ml on Day 6. This corresponded to the glucose depletion for the two cultures, which occurred one day prior to reaching the maximum VCDs (Fig. 3B). As such, by supplementing the 4 g/l glucose culture medium with maltose, we can determine the effects of additional maltose on this non-adapted CHO-K1 production cell line without the added complications of the depletion of other nutrients.

From Fig. 3A, it was observed that all cultures with maltose supplementation have a higher maximum VCD and longer culture viability compared to the 4 g/l glucose only culture, even though the glucose in these culture media were depleted at the same time (Fig. 3B). Recombinant protein production was also maintained in the maltose supplemented cultures to result in a higher IgG titer compared to the 4 g/l glucose only culture (Fig. 3E). When compared to the 6 g/l glucose only culture, the maximum VCD and IgG titers of the maltose supplemented culture were lower (Fig. 3A and G), with the exception of the IgG titer of the 3 g/l maltose supplemented culture, which matched that of the 6 g/l glucose culture. This suggests that while maltose can support cell growth when glucose was depleted before the 6 g/l glucose culture (Fig. 3B), metabolism is slower in the maltose supplemented cultures to result in slower growth rates. This was further supported by the observation that the growth improvement due to maltose was concentration-dependent (Fig. 3A), suggesting that the rate of maltose metabolism may be limiting at these concentrations. This corresponded with the ammonium production profiles too (Fig. 3G), which showed higher ammonium production for the 4 g/l glucose cultures with 0, 0.5 and 1 g/l maltose supplement, despite similar glutamine consumption profiles when compared to the cultures with 6 g/l glucose or that with 3 g/l maltose supplement (Fig. 3F). This suggests that more amino acids are subjected to deamidation to supplement the energy consumption in the cultures with no or low concentrations of maltose. On the other hand, the 3 g/l maltose supplemented culture has a lower ammonia production that was similar to that of the 6 g/l glucose culture (Fig. 3G). This suggests that the rate of maltose metabolism was sufficient for the cells’ reduced energy requirements at this maltose concentration during the later phase of the batch culture.

When lactate profiles are examined, we noted that lactate consumption occurred with the depletion of glucose for all cultures (Fig. 3C). In contrast to glucose-only cultures where lactate consumption was partial, all lactate was consumed at Day 6 for maltose-supplemented cultures. We postulate that the complete lactate consumption may be facilitated by maltose metabolism which kept the culture viable and metabolizing in a low glucose environment for a longer time to utilize the lactate. This property of maltose supplemented cultures may be useful in biopharmaceutical production because lactate accumulation commonly causes cell toxicity in fed-batch bioreactor production processes^15^,^16^. Interestingly, lactate production was observed again from Day 6 onwards for the culture supplemented with 3 g/l maltose, supporting the hypothesis that the maltose metabolism rate was sufficient for the cells to survive on glycolysis at this maltose concentration.

When the culture supernatant maltose concentrations were analyzed, it was observed that maltose was indeed consumed by the cells, and that most consumption occurred when glucose was depleted (Fig. 3D). This suggests that these cells preferentially utilized glucose for growth, and when the glucose was depleted, the culture switched to maltose metabolism which helped to maintain culture viability (Fig. 3A). Interestingly, maltose was depleted at about the same day even though the initial maltose concentrations vary by up to a 6 fold difference. This can be partially attributed to the difference in VCD in the various maltose-supplemented culture, but it also suggests that maltose metabolism may be concentration-dependent at these maltose concentrations, resulting in higher maltose utilization at higher maltose concentrations.

To determine whether maltose could be internalized by the cells, SH87 cell samples from both the 4 g/l glucose-only culture and the 3 g/l maltose-supplemented culture were obtained for LC-MS analysis (Fig. 3H). While intracellular maltose was detected 1 day after seeding into the maltose-supplemented culture medium, it was found to be absent (below detection limits) in cells cultivated in glucose-only medium or immediately after seeding into the maltose-supplemented medium. This confirmed the presence of an intracellular maltose pool in the cultures supplemented with 3 g/l maltose, and demonstrates that maltose did enter these cells, despite the lack of known transport mechanism, adding further evidence that maltose is indeed utilized by the cells in the maltose-supplemented cultures. In addition, further hints regarding maltose transport were noted from the intracellular maltose concentrations, which were maintained at levels approximately 100 times lower than that in the culture medium over the 5 days monitored: This suggests that the transport of maltose may be actively regulated to prevent its accumulation inside the cells despite the concentration gradient, since the cells only switched to maltose metabolism upon glucose depletion (Fig. 3D).

### Maltose is utilized by cells and not hydrolyzed in cell-free media

**Figure 4:**
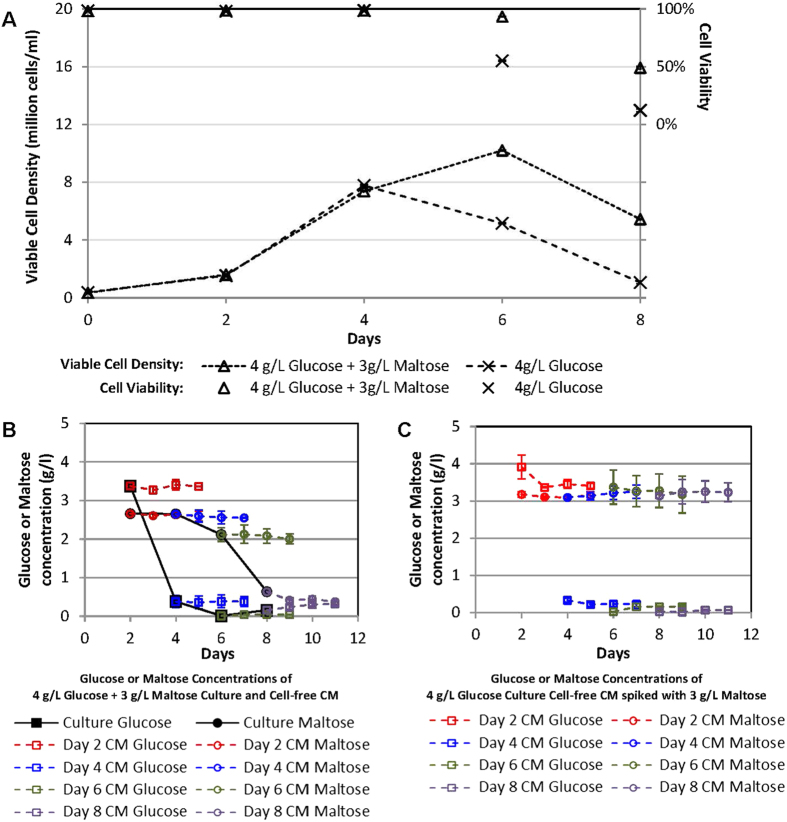
Maltose and glucose profiles in cell-free conditioned media (CM). (**A**) SH87 cell routinely maintained in glucose-only PFCDM was sub-cultivated into PFCDM with 4 g/l glucose, or 4 g/l glucose +3 g/l maltose. On Days 2, 4, 6 and 8, cell-free conditioned medium (CM) were harvested from both cultures. Viable cell densities (lined marker) and culture viabilities (marker only) were also monitored. (**B**) CM from the maltose supplemented culture were incubated for a further 3 days at 37 °C. Samples were harvested daily from these CM, starting from the day of harvest, to determine glucose and maltose concentrations. (**C**) CM harvested from the glucose culture were spiked with maltose to a final concentration of 3 g/l prior to further incubation at 37 °C. Samples from these CM were harvested daily for a further 3 days to determine glucose and maltose concentrations. The averages and standard deviations from 2 replicate shake flasks were plotted.

To further validate that the maltose was indeed utilized by the cells and not hydrolyzed in the culture media, cell-free conditioned media (CM) from Days 2, 4, 6 and 8 were obtained from a 3 g/l maltose supplemented culture as well as a 4 g/l glucose culture. 3 g/l maltose was spiked into the CM from the glucose culture, and both sets of CM were monitored over 3 days for changes in glucose and maltose concentrations at 37 °C (Fig. 4). The growth profiles of the cultures were similar to those in Fig. 3A, with the maltose supplemented culture having a higher viable cell density and extended culture viability at Days 6 and 8 when compared to the glucose culture (Fig. 4A). Glucose and maltose profiles for the maltose supplemented culture were also similar, with glucose depleting on Day 6 and maltose being depleted between Days 4 and 8 (Fig. 4B). On the other hand, the CM harvested from the maltose supplemented culture maintained the same glucose and maltose concentrations at which they were harvested, despite being incubated at the same temperature over 3 days (Fig. 4B). Similarly, the CM from the glucose culture that were spiked with 3 g/l maltose maintained at consistent glucose and maltose concentrations despite the low culture viabilities on Days 6 and 8 (Fig. 4C). This demonstrates that the presence of cells was necessary for the utilization of the maltose, and that maltose hydrolysis was not occurring spontaneously in the conditioned culture media, even when culture viabilities were low. This data suggests that the secreted maltases, such as pancreatic and salivary α-amylases, were not involved in the observed maltose metabolism in these CHO cells. Potential maltases responsible for the observed maltose metabolism are therefore those that are localized with the cells, such as intestinal maltase-glucoamylase and sucrose-isomaltase which are transmembrane proteins, and lysosomal α-glucosidase and neutral α-glucosidase C which are intracellular proteins.

## Discussion

In this study, we demonstrated that mammalian cell lines (CHO-K1, CHO-DG44 and HEK293) can be adapted to grow in maltose-supplemented serum-free protein-free media without glucose. We also demonstrated that an antibody-producing CHO-K1 cell line can utilize maltose for growth and recombinant protein production in a biphasic manner without prior adaptation. We further demonstrated that the supplemented maltose is internalized by the cells and not hydrolyzed in the culture medium. As PFCDM used for the HEK293 adaptation and antibody-producing CHO-K1 culture does not contain serum, hydrolysates nor proteins, we precluded the roles of undefined media components in these observations.

The survival and proliferation of these mammalian cells (3 CHO cell lines and 1 human cell line) in maltose containing protein-free medium is surprising because there is no known mammalian maltose transporter and mammalian cells are typically known to be unable to metabolize disaccharides, unless secreted or transmembrane maltases are expressed, for example in intestinal cells or engineered cell lines. Although CHO cells have been shown to survive using polysaccharides as energy sources, these experiments were performed in serum containing media^7^, and it was demonstrated that serum enzymes break down these polysaccharides for the cells to metabolize^10^. Hence, to our knowledge, this is the first demonstration of a serum-free protein-free mammalian cell culture using a disaccharide as the energy source.

In addition to furthering our knowledge in polysaccharide metabolism in mammalian cells, this discovery presents potential applications since many biopharmaceutical products are produced using serum-free mammalian cell cultures: (1) As maltose metabolism can result in lower lactate production compared to glucose utilization (Fig. 2C), this can be applied to cell culture processes with high lactate issues to improve protein productivity. (2) Maltose supplementation resulted in sustained cell growth and recombinant protein production in batch shake flask cultures (Fig. 3), which can be applied in small scale cultures to facilitate material generation for research and development.

## Methods

### Cell lines and cell cultivation

CHO-K1 cell line was previously adapted to suspension culture in a serum-free protein-free medium, HyQ PF-CHO MPS (Hyclone, Logan, UT) supplemented with 2 g/l sodium bicarbonate (Sigma-Aldrich, St. Louis, MO), 3.6 g/l D-(+)-Glucose (Sigma-Aldrich), 6 mM L-glutamine (Sigma-Aldrich) and 0.1% Pluronic^®^ F-68 (Life Technologies, Carlsbad, CA). Suspension CHO-DG44 cells (Gibco™ Catalog number 12609-012, Invitrogen, Carlsbad, CA) was previously adapted to HyQ PF-CHO (Hyclone, Logan, UT) with 4 mM L-glutamine (Invitrogen), 0.1% Pluronic^®^ F-68 (Invitrogen) and 1× hypoxanthine and thymidine (HT) supplement (Invitrogen). HEK293 cells (CRL-1573, ATCC, Manassas, VA) was previously adapted to a DMEM/F12-based PFCDM supplemented with 6 g/l D-(+)-Glucose (Sigma-Aldrich), 8 mM L-Glutamine (Sigma-Aldrich), and 0.1% Pluronic F-68 (Life Technologies). SH87, a suspension CHO-K1 cell line that is producing an anti-Her2 monoclonal antibody (Ho *et al*.^14^) (courtesy of Dr Yang Yuan Sheng), was previously adapted to a DMEM/F12-based PFCDM supplemented with 6 g/l D-(+)-Glucose (Sigma-Aldrich), 8 mM L-Glutamine (Sigma-Aldrich), 0.1% Pluronic F-68 (Life Technologies), and 600 μg/ml G418 disulfate salt (Sigma-Aldrich). The cells were routinely passaged every 3 to 4 days. Unless otherwise specified, cell cultures in this report were performed in single-use Erlenmeyer flasks (Corning, Acton, MA) incubated in a humidified incubator (Climo-Shaker ISF-1-W, Kuhner, Switzerland) at 37 °C, 8% CO_2_ and a rotation speed of 110 rpm.

### Analysis of cell culture samples

Viable cell density and culture viability were determined by Trypan blue dye exclusion method using Vi-Cell XR Cell Viability Analyzer (Beckman Coulter, Brea, CA) according to manufacturer’s instructions. For biochemical and other cell culture parameter analyses, 1 ml of culture sample was centrifuged at 8000 g for 10 minutes to obtain clarified supernatant. Concentrations of ammonium, glutamine, glucose and lactate were analyzed by the BioProfile 100 Plus (Nova Biomedical, Waltham, MA). Osmolality was measured using a vapor pressure osmometer (Vapro 5520, Wescor, Logan, UT), according to manufacturer’s instructions. Maltose concentration was quantified using Maltose Colorimetric/Fluorometric Assay Kit (Biovision, Milpitas, CA), according to manufacturer’s instructions. Monoclonal IgG antibody titer was determined by nephelometry using IMMAGE 800 (Beckman Coulter), according to manufacturer’s instructions.

### Adaptation of CHO cell lines into culture media with different disaccharides

HyQ PF-CHO disaccharide media were prepared by replacing the 3.6 g/l glucose normally added to the medium, with the same mass concentrations of Maltose (Catalog number M5895, Sigma-Aldrich), Sucrose (Catalog number S1888, Sigma-Aldrich), Lactose (Catalog number L2643, Sigma-Aldrich) or Trehalose (Catalog number T0167, Sigma-Aldrich) during media preparation. CHO-K1 cells were then seeded into these disaccharide media at cell seeding densities of 0.3 × 10^6^ cells/ml and 1.0 × 10^6^ cells/ml. For the adaptation of CHO-DG44 cells, HyQ PF-CHO media were prepared with 10 g/l glucose, maltose, sucrose, lactose or trehalose. CHO-DG44 cells were then seeded into these media at cell seeding densities of 1.0 × 10^6^ cells/ml. For the adaptation of HEK293 cells, PFCDM media were prepared with 10 g/l glucose, maltose, sucrose, lactose or trehalose. HEK293 cells were then seeded into these media at cell seeding densities of 1.0 × 10^6^ cells/ml. The cells were passaged every 3 to 4 days, and viable cell density and culture viability before passage and after inoculation into fresh media were determined at each passage during the adaptation process.

### Shake flask batch cultures sampling and characterization

For growth curve comparison of CHO-K1 cells in maltose and glucose media, CHO-K1 cells adapted to the maltose medium and non-adapted CHO-K1 cells were seeded into HyQ PF-CHO disaccharide medium with 3.6 g/l maltose and the normal HyQ PF-CHO medium with 3.6 g/l glucose, respectively. For the evaluation of maltose utilization using non-adapted SH87, cells were seeded into PFCDM with 4 or 6 g/l glucose, or 4 g/l glucose supplemented with 0.5, 1 or 3 g/l maltose. Cells were cultivated in single-use Erlenmeyer flasks, with a cell seeding density of 0.3 × 10^6^ cells/ml in duplicates. Cell culture samples were collected and analyzed daily throughout the duration of the growth profile experiment until culture viabilities fell below 50%. When necessary, culture supernatant samples were stored at −20 °C for further analysis.

### Quantification of intracellular maltose

Samples for intracellular maltose quantification were prepared by first quenching 10^7^ cells with ice cold 150 mM NaCl (Merck, ACS Reagent Grade) solution. After the cells were pelleted by centrifugation, the supernatant was removed by aspiration and 10 μl of 0.4 mM 13C-maltose (UL-^13^C_12_ maltose monohydrate, Omicron Biochemicals, South Bend, IN) was added as a reference standard. A two-phase liquid extraction protocol, involving the use of methanol (Fisher Scientific, Optima grade), chloroform (Merck) and tricine (Merck) solution (40:35:25 v/v)^17^ was utilized to extract intracellular maltose. The extracts were stored at −80 °C, dried under vacuum at a temperature of 4 °C (CentriVap, Labconco, US) and reconstituted in a water-methanol mixture (95:5 v/v) before analysis via liquid chromatography-mass spectrometry (LC-MS) (Acquity UPLC-Xevo TQ-S MS, Waters, Milford, MA). The separation was performed using a C18 reverse phase column (Waters, HSS T3 column, 2.1 mm × 50 mm, 1.8 μm particle size), with the following solvents - A: water with 0.1% formic acid (Sigma-Aldrich, 98%), B: Methanol, at a flow rate of 0.4 ml/min. Quantification of intracellular maltose was carried out via multiple reaction monitoring experiments, in which the integrated peak areas for maltose and 13C-maltose in each sample were obtained. The actual concentration of maltose in each sample was quantified by direct comparison of the relative integrated peak area of maltose to that of the 13C-maltose reference standard. The lower limit of detection for maltose was observed to be 7.5 ng per injection. Intracellular maltose concentration was calculated based on maltose quantities within detection limit, number of cells used and average cell diameter obtained from Vi-Cell XR Cell Viability Analyzer. The samples were analyzed on LC-MS in triplicate and the integrated peak areas obtained for each sample were observed to have relative standard deviations of less than 15%.

### Calculation

The cumulative integrated viable cell density (IVCD) was calculated by trapezium rule according to Equation 1, where VCD is the viable cell density and t is the culture time.


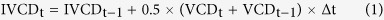


## Additional Information

**How to cite this article:** Leong, D. S. Z. *et al*. Evaluation and use of disaccharides as energy source in protein-free mammalian cell cultures. *Sci. Rep.* **7**, 45216; doi: 10.1038/srep45216 (2017).

**Publisher's note:** Springer Nature remains neutral with regard to jurisdictional claims in published maps and institutional affiliations.
